# Simultaneous RNA quantification of human and retroviral genomes reveals intact interferon signaling in HTLV-1-infected CD4+ T cell lines

**DOI:** 10.1186/1743-422X-9-171

**Published:** 2012-08-23

**Authors:** Britta Moens, Christophe Pannecouque, Giovanni López, Michael Talledo, Eduardo Gotuzzo, Ricardo Khouri, Achiléa Bittencourt, Lourdes Farré, Bernardo Galvão-Castro, Anne-Mieke Vandamme, Johan Van Weyenbergh

**Affiliations:** 1 Laboratory of Clinical and Epidemiological Virology, Rega Institute for Medical Research, K.U.Leuven, Leuven, Belgium; 2 Laboratory for Virology and Chemotherapy, Rega Institute for Medical Research, K.U.Leuven, Leuven, Belgium; 3 Instituto de Medicina Tropical Alexander von Humboldt, Universidad Peruana Cayetano Heredia, Lima, 31, Peru; 4 Departamento de Medicina, Facultad de Medicina, Universidad Peruana Cayetano Heredia, Lima, Peru; 5 Gonçalo Moniz Research Center, Oswaldo Cruz Foundation (FIOCRUZ), Salvador-Bahia, Brazil; 6 Department of Pathology, Professor Edgar Santos Teaching Hospital, Federal University of Bahia, Salvador-Bahia, Brazil; 7 Bahia School of Medicine and Public Health, Salvador-Bahia, Brazil; 8 Centro de Malária e outras Doenças Tropicais, Instituto de Higiene e Medicina Tropical, Universidade Nova de Lisboa, Lisboa, Portugal; 9 Institute for Immunological Investigation iii-INCT, Sao Paulo, Brazil

**Keywords:** Retrovirus, IFN-α, HIV-1, HTLV-1, IFN-α signaling, Antiviral activity

## Abstract

**Background:**

IFN-α contributes extensively to host immune response upon viral infection through antiviral, pro-apoptotic, antiproliferative and immunomodulatory activities. Although extensively documented in various types of human cancers and viral infections, controversy exists in the exact mechanism of action of IFN-α in human immunodeficiency virus type 1 (HIV-1) and human T-lymphotropic virus type 1 (HTLV-1) retroviral infections.

**Results:**

IFN-α displayed strong anti-HIV-1 effects in HIV-1/HTLV-1 co-infected MT-4 cells in vitro, demonstrated by the dose-dependent inhibition of the HIV-1-induced cytopathic effect (IC_50_ = 83.5 IU/ml, p < 0.0001) and p24 levels in cell-free supernatant (IC_50_ = 1.2 IU/ml, p < 0.0001). In contrast, IFN-α treatment did not affect cell viability or HTLV-1 viral mRNA levels in HTLV-1 mono-infected cell lines, based on flow cytometry and nCounter analysis, respectively. However, we were able to confirm the previously described post-transcriptional inhibition of HTLV-1 p19 secretion by IFN-α in cell lines (p = 0.0045), and extend this finding to primary Adult T cell Leukemia patient samples (p = 0.031). In addition, through microarray and nCounter analysis, we performed the first genome-wide simultaneous quantification of complete human and retroviral transciptomes, demonstrating significant transcriptional activation of interferon-stimulated genes without concomitant decrease of HTLV-1 mRNA levels.

**Conclusions:**

Taken together, our results indicate that both the absence of in vitro antiproliferative and pro-apoptotic activity as well as the modest post-transcriptional antiviral activity of IFN-α against HTLV-1, were not due to a cell-intrinsic defect in IFN-α signalisation, but rather represents a retrovirus-specific phenomenon, considering the strong HIV-1 inhibition in co-infected cells.

## Background

The two pathogenic human retroviruses, human immunodeficiency virus type 1 (HIV-1) and human T-lymphotropic virus type 1 (HTLV-1), remain responsible for significant morbidity and mortality worldwide. At present, 33 million people are estimated to be infected with HIV-1 and 10 to 30 million people with HTLV-1 [1,2]. Although both retroviruses share similarities in routes of transmission and in vivo tropism for CD4+ T cells, HIV-1 and HTLV-1 significantly differ in pathogenicity, disease progression and treatment outcome. Current treatment of HIV-1-infected individuals is based on highly active antiretroviral therapy (HAART), combining HIV-1 reverse transcriptase, protease, integrase and/or entry inhibitors [3]. HAART reduces HIV-1 plasma viral loads to undetectable levels in the majority of patients, hereby slowing down clinical progression to the acquired immunodeficiency syndrome (AIDS). HTLV-1, on the other hand, is able to cause adult T cell leukemia/lymphoma (ATLL), as well as HTLV-1 associated myelopathy/tropical spastic paraparesis (HAM/TSP), albeit in a minority of infected individuals [1,4,5]. ATLL is an aggressive T cell malignancy of mainly CD4 + CD25+ T cells, affecting up to 6% of HTLV-1-infected individuals [5]. The diversity of patient symptoms together with their prognosis has led to the classification of ATLL into four clinical subtypes: acute, lymphoma, smouldering and chronic ATLL. Recently, consensus guidelines have been drafted for ATLL treatment with first line therapy comprising combination chemotherapy regimens for lymphoma patients and zidovudine (AZT) and interferon-α (IFN-α) combination therapy for treatment of acute, smouldering and chronic ATLL patients [6,7]. Up to 5% of HTLV-1-infected individuals may develop HAM/TSP, a neuro-inflammatory disease characterized by demyelinating lesions and lymphocytic activation and infiltration into the central nervous system [8,9]. Treatment of HAM/TSP patients consists of mainly symptomatic (antispasmodics) and empirical strategies such as the use of immunosuppressive drugs (corticosteroids), IFN-α or vitamin C [10]. However, consensus guidelines for HAM/TSP treatment are missing due to the lack of randomized controlled clinical trials.

Although recent genetic evidence has implicated IFN/STAT1/FAS signaling in both HAM/TSP and ATL pathogenesis [11,12], therapeutic options include IFN-α for both diseases. Initially, IFN-α was implemented in anti-HTLV-1 treatment because of its effect in HIV-1/AIDS treatment [13]. IFN-α was one of the first cytokines to be discovered and contributes extensively to the host immune response upon viral infection. Type I interferons (IFN-α/β) are expressed and secreted by various cell types in response to viral infection or recognition of pathogens [14-16]. IFN-α signaling pathways are initiated by binding of IFN-α to the heterodimeric interferon-α/β receptor, consecutively activating JAK/STAT pathways. Phosphorylated STAT1 homodimers and/or the interferon-stimulated gene factor 3 (ISGF3) complex, comprising phosphorylated STAT1, phosphorylated STAT2 and interferon regulatory factor 9 (IRF9), are formed, leading to the transcriptional activation of interferon-stimulated genes (ISGs) [17,18]. Classical well-characterized ISGs include 2’,5’-oligoadenylate synthetase (OAS), protein kinase R (PKR), interferon regulatory factors (IRFs) and myxovirus resistance (Mx) genes [15,17,19]. Although the precise function of several other ISGs remains poorly understood, expression of ISGs inhibits replication of various types of viruses [19-23]. In addition to antiviral activity, IFN-α has profound effects on apoptosis and immune response in several types of human cancer and viral infections [16,24-31].

HIV-1 replication can be inhibited in vitro by IFN-α in both macrophages as well as T cells, affecting both early and late stages of the viral replication cycle [20,32-38]. Antiviral activity of IFN-α was characterized by the expression of numerous ISGs. However, the in vivo antiviral activity of IFN-α and its contribution to HIV-1 disease progression remain controversial and need to be further elucidated. Higher levels of serum IFN-α, IFN-α-induced immune activation and higher transcriptional levels of ISGs are paradoxically correlated with higher HIV-1 viral load in HIV-1-infected individuals [39-43], as well as disease progression, questioning its antiviral activity in vivo. Nevertheless, clinical trials have reported modest but significant decreases in HIV-1 viral load after IFN-α treatment [13,44-46].

In HTLV-1-infected cells, in vitro antiviral activity has been reported for IFN-α by a post-transcriptional mechanism impairing viral assembly. Hereby, IFN-α had no effect on viral protein synthesis, but inhibited virion release through the inhibition of Gag protein association with lipid rafts [47]. In addition, IFN-α stimulation induced transcriptional activation of ISGs in HTLV-1-infected cells, reflecting intact IFN-α signaling pathways [48]. However, HTLV-1 expression has been reported to blunt IFN-α signaling pathways in vitro via HTLV-1 Tax and suppressor of cytokine signaling 1 (SOCS1) expression [48-50]. Such inhibition could impair antiviral, immunomodulatory and antiproliferative activities of IFN-α in vivo. Nevertheless, significant clinical benefit has been demonstrated for ATLL patients treated with AZT and IFN-α combination therapy and HAM/TSP patients treated with IFN-α monotherapy [7,51,52]. In conclusion, although IFN-α signaling pathways contribute extensively to the host immune and antiviral responses and therefore significantly determine the therapeutic success of IFN-α treatment, controversy exists in the exact mechanism of action of IFN-α in retroviral infections.

In the present study, we address the possible antiviral, antiproliferative and/or pro-apoptotic effects of IFN-α in HTLV-1 mono-infected and HIV-1/HTLV-1 co-infected CD4+ T cell lines in vitro. We demonstrate that IFN-α exerts pronounced anti-HIV-1 effects in HTLV-1 co-infected cells, but has a limited, post-transcriptional, antiviral effect upon HTLV-1 in mono-infected cells. As demonstrated by gene expression profiling, both STAT1/STAT2-mediated antiviral signaling pathways and broad ISGs induction were fully activated by IFN-α in HTLV-1-infected cells. However, IFN-α failed to inhibit mRNA levels of any of the known HTLV-1 genes or to induce significant pro-apoptotic or antiproliferative activity in HTLV-1-infected cells. In conclusion, the apparent absence of IFN-α antiretroviral activity is selective towards HTLV-1, since a significant anti-HIV-1 effect can be observed in co-infected cells.

## Results

### IFN-α selectively affects cell viability of HIV-1/HTLV-1 co-infected but not HTLV-1 mono-infected cells

**Figure 1:**
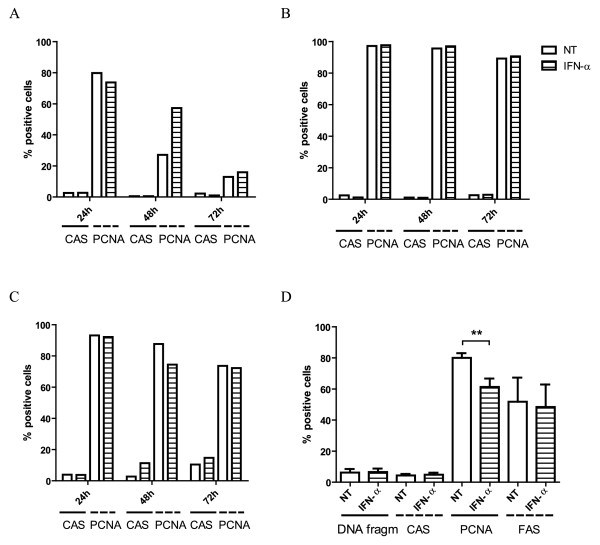
**Flow cytometric analysis of cell death- and cell proliferation-associated markers of HTLV-1-infected cell lines. **HTLV-1-infected CD4+ T cell lines, (**A**) C8166, (**B**) MT-2 and (**C**) MT-4 were treated for 24, 48 and 72 hours with no treatment (NT) or IFN-α, illustrated here for a fixed concentration of 1000 IU/ml. The percentage of active-caspase 3-positive cells (CAS) and proliferating cell nuclear antigen-positive cells (PCNA) were determined by flow cytometry and shown in the *y*-axis. The time points of treatment together with the treatment conditions are shown in the *x*-axis. (**D**) MT-4 cells were treated for 48 hours with no treatment (NT) or IFN-α (1000 IU/ml) in six independent experiments. Flow cytometric quantification of cells with DNA fragmentation (Hoechst 33342-positive cells), active-caspase 3-positive cells (CAS), proliferating cell nuclear antigen-positive cells (PCNA) and Fas receptor-positive cells (FAS) are shown in the *y*-axis, whereas the treatment conditions are shown in the *x*-axis. Parametric t-tests were used with p-values indicated by asterisks (^*^ < 0.05, ^**^ < 0.01 and ^***^ < 0.001).

To assess the effects of IFN-α on cell viability of HTLV-1-infected cell lines, we quantified pro-apoptotic (active-caspase 3) and proliferation-associated (PCNA) markers via flow cytometry after IFN-α treatment (10 - 10^3^ IU/ml) at different time points. As illustrated in Figure 1, IFN-α had no marked effect upon active-caspase 3-positive cells in three different HTLV-1-infected CD4+ T cell lines (C8166, MT-2 and MT-4) at 24, 48 and 72 hours of treatment. C8166 cells showed a spontaneous decline in proliferation (Figure 1A), reflected by pronounced time-dependent reduction in the percentage of PCNA-positive cells, making them unsuitable for long-term experiments (> 72 hours), which were further on carried out only with MT-2 and MT-4 cells. We observed a modest, but significant, decrease in the percentage of PCNA-positive MT-4 cells by 18.7 ± 5.4% (n = 6, t-test, p = 0.006), after 48 hours of treatment (Figure 1C–D). Quantification of additional cell death-associated markers (DNA fragmentation, surface Fas) revealed no pro-apoptotic activity of IFN-α in MT-4 cells after 48 hours of treatment (Figure 1D). Nevertheless, both MT-2 and MT-4 cells were resistant to IFN-α after 72 hours (Figure 1B–C) and even in long-term culture (15 days, data not shown), with similar cell viability in comparison to untreated cells.

**Figure 2:**
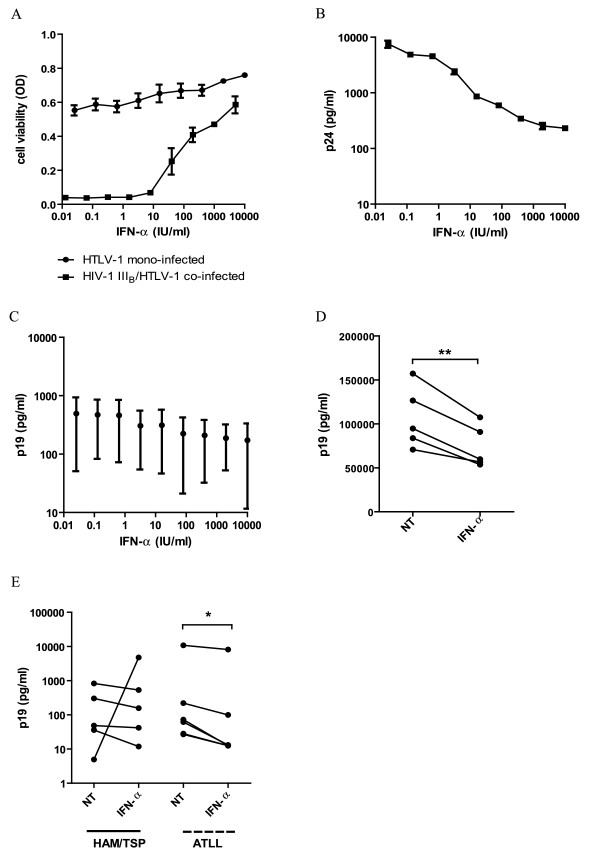
**Cell viability, HIV-1 p24 and HTLV-1 p19 levels of HTLV-1 co- and mono-infected cells. **HIV-1 III_B_/HTLV-1 co-infected and HTLV-1 mono-infected MT-4 cells were cultured in the absence or presence of varying concentrations of IFN-α (0 - 10^4^ IU/ml). (**A**) Cell viability was determined after 5 days of culture, based on reduction of MTT and expressed in optical density (OD). In parallel, (**B**) HIV-1 p24 protein and (**C**) HTLV-1 p19 protein concentrations (pg/ml), were determined in cell-free supernatant of HIV-1/HTLV-1 co-infected and HTLV-1 mono-infected MT-4 cells, respectively, after 48 hours of culture. In addition, (**D**) HTLV-1 positive MT-2 cells were treated for 48 hours with no treatment (NT) or a fixed concentration of 1000 IU/ml of IFN-α, followed by quantification of HTLV-1 p19 protein levels in cell-free supernatant. (**E**) HTLV-1 p19 protein levels were also determined in cell-free supernatant of PBMCs of HAM/TSP (n = 5) and ATLL (n = 6) patients, after 48-72 hours of culture in the absence or presence of IFN-α (1000 IU/ml). Parametric t-tests were used with p-values indicated by asterisks (^*^ < 0.05, ^**^ < 0.01 and ^***^ < 0.001).

Using an established MTT assay [53], we assessed the effects of IFN-α on cell viability of HIV-1 III_B_/HTLV-1 co-infected MT-4 cells and HTLV-1 mono-infected MT-4 cells (HIV-1 negative, HTLV-1 positive). After 5 days of in vitro stimulation with varying concentrations of IFN-α (10 - 10^4^ IU/ml), inhibition of the HIV-1-induced cytopathic effect (CPE) on MT-4 cells was measured and inhibitory concentration (IC_50_) was calculated. IFN-α strongly inhibited the HIV-1-induced CPE on the co-infected MT-4 cells with a mean IC_50_ of ± 83.5 IU/ml (n = 3, area under curve, p < 0.0001, 2519 ± 91.22 versus 7316 ± 115.2, Figure 2A). In contrast, cell viability of HTLV-1 mono-infected MT-4 cells was not affected by IFN-α treatment as demonstrated by a mean cytotoxic concentration (CC_50_) higher than 10^4^ IU/ml (highest concentration tested, n = 3, Figure 2A), supporting flow cytometric observations in MT-4 cells.

### IFN-α induces a 1000-fold greater reduction of HIV-1 p24 vs. HTLV-1 p19 protein levels

Although IFN-α strongly induced anti-HIV-1 activity in MT-4 cells, we observed no “anti-HTLV-1” activity of IFN-α in MT-4 cells based on cell viability data (MTT reduction and flow cytometry). Since HIV-1 p24 and HTLV-1 p19 levels are *bona fide* readouts of virion production, we quantified these viral proteins in cell-free supernatant of HIV-1 III_B_/HTLV-1 co-infected and HTLV-1 mono-infected MT-4 cells, respectively, treated with increasing concentrations of IFN-α for 48 hours (n = 3). IFN-α dose-dependently reduced HIV-1 p24 levels (ANOVA, p < 0.0001, Bonferroni post-test, p < 0.05, significant from 0.64 IU/ml), with a mean IC_50_ of 1.2 IU/ml and thus reflecting strong inhibition of HIV-1 p24 production and/or secretion in HIV-1/HTLV-1 co-infected MT-4 cells (Figure 2B). In parallel, IFN-α did not markedly affect HTLV-1 p19 levels in cell-free supernatant of HTLV-1 mono-infected MT-4 cells (ANOVA, p = 0.96), even at high doses (Figure 2C). In addition, HTLV-1 p19 protein levels were also determined in cell-free supernatant of the HTLV-1 positive and virion-producing MT-2 cell line. To obtain maximum biological activity, MT-2 cells were treated for 48 hours with a fixed concentration of 1000 IU/ml of IFN-α. IFN-α significantly reduced p19 levels in cell-free supernatant of MT-2 cells by 31% (n = 5, t-test, p = 0.0045, Figure 2D), confirming the results of Feng *et al.*[47]. Nevertheless, IFN-α was 1,000-fold more active against HIV-1 than HTLV-1, when judged according to the inhibition of Gag protein levels (p24 for HIV-1 and p19 for HTLV-1). In HIV-1 III_B_/HTLV-1 co-infected MT-4 cells, 1.2 IU/ml of IFN-α inhibited 50% of HIV-1 p24 levels, whereas 1000 IU/ml of IFN-α was not even sufficient to inhibit 50% of HTLV-1 p19 levels in HTLV-1 mono-infected cells.

To evaluate the potential clinical relevance of this observation, we quantified HTLV-1 p19 levels in cell-free supernatant of HAM/TSP (n = 5) and ATLL (n = 6) PBMCs. IFN-α exerted no significant effect upon p19 levels of HAM/TSP PBMCs (*t*-test, p = 0.63), in agreement to our recent demonstration of a lack of *ex vivo* antiviral, pro-apoptotic and antiproliferative effects of IFN-α in HAM/TSP patients [54]. However, IFN-α significantly decreased p19 levels of ATLL PBMCs with 26% (*t*-test, p = 0.031) (Figure 2E). This modest reduction of p19 levels observed in ATLL PBMCs was highly similar to the IFN-α-induced reduction observed in MT-2 cells (Figure 2D) and to recent findings in larger cohorts of HTLV-1-infected individuals [55] as well as ATL patients (Khouri *et al.*, unpublished).

### Gene expression profiling of IFN-α-treated HTLV-1-infected cell lines

While HIV-1 replication was extremely sensitive to IFN-α antiviral activity in HIV-1/HTLV-1 co-infected MT-4 cells, anti-HTLV-1 activity remains questionable since we did not detect an effect of IFN-α on cell viability of HTLV-1 mono-infected cells. However, we could measure a modest inhibitory effect of IFN-α on HTLV-1 p19 protein levels in MT-2 cells. To clarify whether IFN-α signaling is blunted in HTLV-1-infected cell lines, possibly explaining the lack of IFN-α responsiveness, we performed gene expression profiling of MT-2 and MT-4 cells after 6 hours of IFN-α stimulation. Agilent Whole Humane Genome microarray analysis was performed in two separate microarray experiments with duplicate samples for both cell lines. Gene expression profiling of MT-2 revealed 77 genes significantly regulated by IFN-α, of which 64 were up- and 13 down-regulated. Gene expression profiling of MT-4 revealed 284 genes significantly regulated by IFN-α, of which 262 were up- and 22 down-regulated. The top 20 of the most significant up-regulated genes are shown in Table 1 for MT-2 and Table2 for MT-4 cells, and included classical ISGs such as Mx, OAS and IRFs genes. We found no common down-regulated genes for MT-2 and MT-4, while 69% (44/64) of up-regulated genes of MT-2 were also up-regulated in MT-4 cells. Altogether, gene expression profiling demonstrated significant activation of ISGs and thus intact IFN-α signaling in HTLV-1-infected MT-2 and MT-4 cells.

**Table 1:** Overview of the 20 most significant up-regulated genes by IFN-α treatment in MT-2 cells

***Gene Symbol***	***GAN***	***Gene Name***
MX1	NM_002462	myxovirus (influenza virus) resistance 1 (mouse)
DTX3L	NM_138287	deltex 3-like (Drosophila)
IFIT5	NM_012420	interferon-induced protein with tetratricopeptide repeats 5
TMEM140	NM_018295	transmembrane protein 140
BCL2L14	NM_030766	BCL2-like 14 (apoptosis facilitator)
EIF2AK2	NM_002759	eukaryotic translation initiation factor 2-alpha kinase 2
SP100	NM_001080391	SP100 nuclear antigen
EPSTI1	AL831953	epithelial stromal interaction 1 (breast)
RTP4	NM_022147	receptor (chemosensory) transporter protein 4
RSAD2	NM_080657	radical S-adenosyl methionine domain containing 2
IRF7	NM_004031	interferon regulatory factor 7
TRIM21	NM_003141	tripartite motif-containing 21
IFIH1	NM_022168	interferon induced with helicase C domain 1
TRIM25	NM_005082	tripartite motif-containing 25
C19orf66	NM_018381	chromosome 19 open reading frame 66
PARP9	NM_031458	poly (ADP-ribose) polymerase family, member 9
PML	NM_033244	promyelocytic leukemia
OAS3	NM_006187	2'-5'-oligoadenylate synthetase 3, 100 kDa
IFI44	NM_006417	interferon-induced protein 44
XAF1	NM_017523	XIAP associated factor 1

GAN: Genbank Accession Number.

**Table 2:** Overview of the 20 most significant up-regulated genes by IFN-α treatment in MT-4 cells

***Gene Symbol***	***GAN***	***Gene Name***
IFIT1	NM_001548	interferon-induced protein with tetratricopeptide repeats 1
STAT1	NM_007315	signal transducer and activator of transcription 1, 91 kDa
ADAR	NM_001111	adenosine deaminase, RNA-specific
IFIT5	NM_012420	interferon-induced protein with tetratricopeptide repeats 5
IRF1	NM_002198	interferon regulatory factor 1
APOL6	NM_030641	apolipoprotein L, 6
MX2	NM_002463	myxovirus (influenza virus) resistance 2 (mouse)
HAPLN3	NM_178232	hyaluronan and proteoglycan link protein 3
EIF2AK2	NM_002759	eukaryotic translation initiation factor 2-alpha kinase 2
BCL2L14	NM_030766	BCL2-like 14 (apoptosis facilitator)
LAP3	NM_015907	leucine aminopeptidase 3
IRF7	NM_004029	interferon regulatory factor 7
ZBTB32	NM_014383	zinc finger and BTB domain containing 32
SP100	NM_001080391	SP100 nuclear antigen
EPSTI1	NM_033255	epithelial stromal interaction 1 (breast)
TBX21	NM_013351	T-box 21
SAMD9L	NM_152703	sterile alpha motif domain containing 9-like
IFI27	NM_005532	interferon, alpha-inducible protein 27
TAP1	NM_000593	transporter 1, ATP-binding cassette, sub-family B (MDR/TAP)
TNFSF13B	NM_006573	tumor necrosis factor (ligand) superfamily, member 13b

GAN: Genbank Accession Number.

### Pathway analysis confirms intact IFN-α antiviral signaling pathways in HTLV-1-infected cell lines

**Figure 3:**
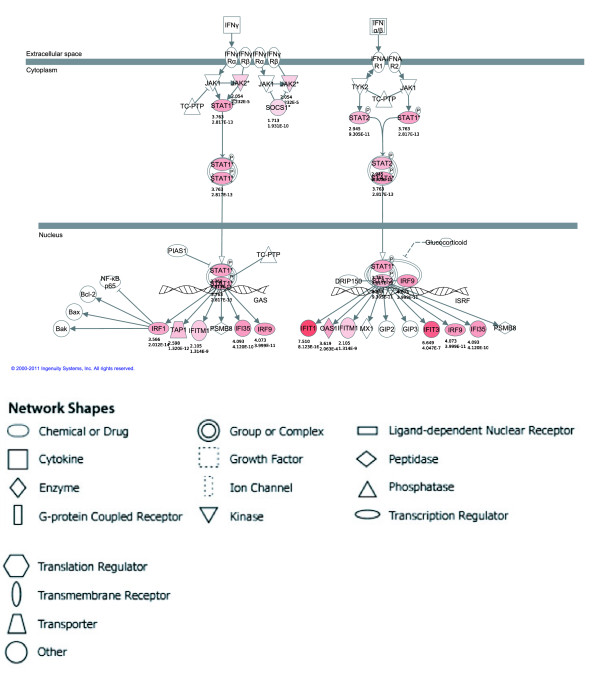
**The principal IFN-α-modulated canonical pathway in MT-4 cells. **Ingenuity Pathway Analysis (IPA) was performed on genes regulated by IFN-α treatment (6 h) in MT-2 (**A**) and MT-4 cells (**B**). The principal canonical pathway, interferon signalling, is graphically displayed for MT-4 only (**C**). The red colour indicates up-regulation with the intensity indicating the degree of the gene transcription change. The log_2_ fold-change values and p-values are indicated below each molecule. Full lines represent direct interactions, while dashed lines indirect interactions. Network shapes are represented in the legend.

In order to characterize possible antiviral, pro-apoptotic and/or antiproliferative pathways represented by the respective IFN-α up- and down-regulated genes, which were identified via microarray analysis described above, Ingenuity Pathway Analysis (IPA) was performed for both cell lines. Genes were sorted into molecular gene networks and canonical pathways, of which significantly overrepresented networks/pathways were identified. In the case of MT-2, seven molecular networks were identified as significantly modulated by IFN-α treatment. The principal network contained 25 genes, all up-regulated, representing antimicrobial and inflammatory responses. In the case of MT-4, 12 molecular networks could be identified as significantly modulated by IFN-α treatment. The principal network contained 26 genes, all up-regulated, representing infection mechanism. The principal IFN-α-modulated canonical pathway was identical for both cell lines, *i.e.* interferon signaling (Figure 3), although subtle differences were observed (IFN-γ, MX1, up-regulated only in MT-2 and JAK2, SOCS1, IRF1, TAP1, OAS1, up-regulated only in MT-4). Thus, IPA illustrated IFN-α-induced modulation of molecular networks and pathways implicated in viral infection and/or IFN signaling in both cell lines. Moreover, no apoptotic, cell cycle-related or antiproliferative pathways were identified, corroborating our flow cytometry findings that IFN-α had no effect on cell viability of HTLV-1-infected cell lines.

### Lack of antiviral activity of IFN-α in HTLV-infected cell lines

**Figure 4:**
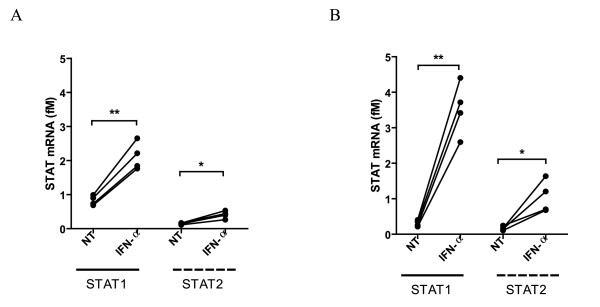
**Quantification of STAT1 and STAT2 mRNA levels of HTLV-1-infected cell lines.** HTLV-1-infected CD4+ T cell lines (**A**) MT-2 and (**B**) MT-4 were treated for 6 hours with no treatment (NT) or IFN-α (1000 IU/ml). mRNA levels (fM) of cellular STAT1 and STAT2 genes were assessed via nCounter analysis and shown in the *y*-axis, whereas the treatment conditions are shown in the *x*-axis. Parametric t-tests were used with p-values indicated by asterisks (^*^ < 0.05, ^**^ < 0.01 and ^***^ < 0.001).

**Figure 5:**
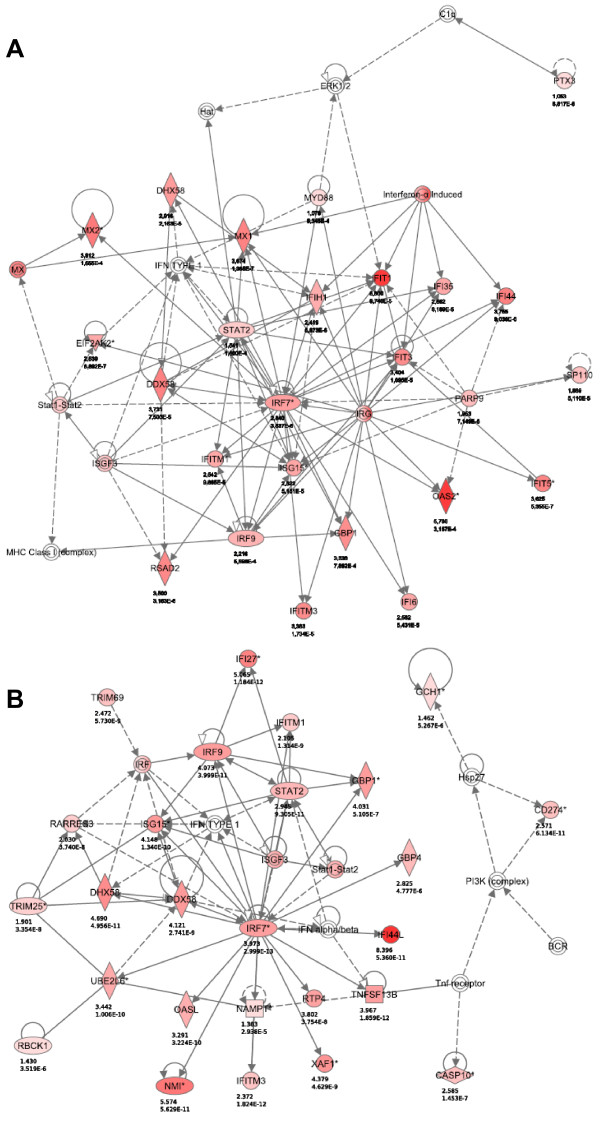
**STAT1/STAT2 signaling pathways predominate following IFN-α treatment in MT-2 and MT-4 cells. **Ingenuity Pathway Analysis (IPA) was performed on genes regulated by IFN-α treatment (6 h) in MT-2 (**A**) and MT-4 cells (**B**). The red colour indicates up-regulation with the intensity indicating the degree of the gene transcription change. The log_2_ fold-change values and p-values are indicated below each molecule. Full lines represent direct interactions, while dashed lines indirect interactions. Network shapes are as in Figure 3.

**Figure 6:**
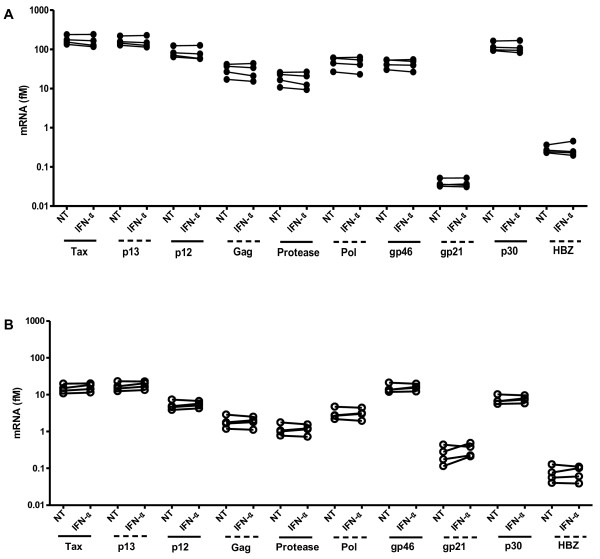
**Quantification of HTLV-1 viral mRNA levels after 6 hours of IFN-α treatment. **HTLV-1-infected CD4+ T cell lines (**A**) MT-2 and (**B**) MT-4 were treated for 6 hours with no treatment (NT) or IFN-α (1000 IU/ml). mRNA levels (fM) of all known HTLV-1 viral genes were assessed via nCounter analysis and shown in the *y*-axis, whereas the treatment conditions are shown in the *x*-axis, represented for each of the viral genes. No statistical significance was observed for any gene (t-test).

To assess the possible antiviral activity of IFN-α against HTLV-1 and to validate cellular genes identified through microarray analysis, nCounter analysis was performed [56]. All known HTLV-1 viral genes, including the antisense HBZ, and a selection of specific cellular genes, *i.e.* STAT1, STAT2, STAT3 and CD69, an IFN-induced early activation marker of T lymphocytes, were included for nCounter analysis. mRNA levels of selected genes were quantified and normalized to a cellular housekeeping gene (hypoxanthine phosphoribosyl-transferase 1). IFN-α significantly increased mRNA levels of STAT1 and STAT2 genes in MT-2 and MT-4 cells (n = 4, *t*-test, p = 0.0024, p = 0.010 and p = 0.0023, p = 0.032, respectively, Figure 4), confirming functional activation of JAK/STAT pathways and IFN-α antiviral signaling. Moreover, mRNA levels of cellular STAT3 and CD69 genes were also significantly increased in MT-4 cells, confirming our microarray results (n = 4, t-test, p = 0.0041, p = 0.0018, respectively, data not shown). As indicated by pathway analysis (Figure 5A-B), IFN-α predominantly activated STAT1 and STAT2 signaling and downstream genes, representing main antiviral pathways, rather than STAT3 signaling, representing inflammatory and carcinogenic pathways. HTLV-1 viral mRNA levels were ± 10-fold higher in MT-2 cells, in comparison to MT-4 cells, in agreement with the superior virion production by MT-2 cells (Figure 2C-D). Surprisingly, after 6 hours, IFN-α treatment had no effect on mRNA levels of any of the HTLV-1 viral genes in both cell lines (n = 4, *t*-test, Figure 6A-B). Due to the surprising absence of an antiviral effect at 6 hours, we also performed nCounter analysis after 2 and 48 hours of IFN-α treatment of MT-2 and MT-4 cells to verify whether “early” or “late” inhibition of HTLV-1 viral gene transcription might occur. At 2 hours, IFN-α again had no effect on viral gene mRNA levels in both cell lines (n = 4, *t*-test, data not shown). However, IFN-α significantly increased mRNA levels of STAT1 and STAT2 genes in MT-4 cells after 2 hours (n = 4, *t*-test, p = 0.023 and p = 0.029, respectively, Additional file 1), illustrating early activation of JAK/STAT pathways and IFN-α signaling. At 48 hours, IFN-α again failed to considerably down-regulate HTLV-1 viral gene transcription (Additional file 1) although IFN-α signaling was sustained in both MT-2 and MT-4 cells, demonstrated by increased STAT1 and STAT2 mRNA levels (Additional file 1). Altogether, IFN-α treatment induced intact and sustained IFN-α signaling in HTLV-1-infected cell lines, without any antiviral activity at the transcriptional level, as all HTLV-1 viral mRNA levels were unaffected after 2, 6 and 48 hours of IFN-α treatment.

## Discussion

IFN-α treatment exerted a strong in vitro anti-HIV-1 effect in HIV-1/HTLV-1 co-infected MT-4 cells, demonstrated by the dose-dependent inhibition of the HIV-1-induced CPE and p24 secreted levels upon HIV-1 infection. In contrast, IFN-α treatment did not affect cell viability of HTLV-1-infected cell lines C8166, MT-2 nor MT-4. Furthermore, we demonstrate for the first time that IFN-α treatment did not affect HTLV-1 viral mRNA levels in MT-2 and MT-4 cells, demonstrated by nCounter analysis at various time points. Although these observations suggest the lack of biological activity of IFN-α against HTLV-1, we were able to confirm the previously described post-transcriptional inhibition of HTLV-1 p19 secretion by IFN-α [47], both in cell lines as well as in ATL patient samples. However, in comparison to the strong dose-dependent inhibition of HIV-1 p24 levels, IFN-α only modestly reduced p19 levels in cell-free supernatant of MT-2 cells and ATL PBMCs. Despite the absence of pronounced antiviral, pro-apoptotic and antiproliferative activity of IFN-α, microarray and nCounter analysis revealed significant transcriptional activation of ISGs and intact IFN-α signaling in MT-2 and MT-4 cells. MT-4 cells appeared to be more responsive to IFN-α treatment in comparison to MT-2 cells, demonstrated by significant up- or down-regulation of 284 vs. 77 genes, respectively, and the early activation of STAT1 and STAT2 genes. Nevertheless, approximately 70% of the IFN-α-up-regulated genes in MT-2 were identical of those up-regulated in MT-4 cells, indicating the similarity and reliability of both cell lines as HTLV-1 in vitro models.

Because of the intact transcriptional activation of ISGs, the absence of distinct antiviral activity of IFN-α against HTLV-1 was not due to a general defect in IFN-α signaling pathways in MT-2 or MT-4 cells. In agreement with our findings, over-expression of a subset of ISGs in chronic HTLV-1 infection has recently been shown to fail to constitute an efficient antiviral host response, but might instead contribute to HAM/TSP pathogenesis [55]. We speculate that IFN-α fails to decrease HTLV-1 mRNA levels due to highly virus-specific retroviral restriction factors, as IFN-α exerted strong anti-HIV-1 yet weak anti-HTLV-1 effects in HIV-1/HTLV-1 co-infected MT-4 cells. Since all known HTLV-1 mRNAs, including antisense HBZ, remain unchanged upon IFN treatment, defective RNAseL activity, downstream of OAS gene activation (reviewed in [57]), might be hypothesized as a possible HTLV-1 escape mechanism,. Although several OAS family members are IFN-inducible in both HTLV-1 infection (this study, Table I and results not shown) and HIV-1 infection [40], little is known of downstream RNAse L activation, which occurs at the protein level [57]. In addition, blunting of IFN-α biological activity has been mainly addressed in HTLV-1 mono-infection, but not in HIV-1/HTLV-1 coinfection. HTLV-1 expression has been reported to up-regulate SOCS1 expression, inducing ubiquitination and proteasomal degradation of IRF3, leading to the inhibition of type I interferon production and thus inhibiting activation of IFN-α signaling pathways [50]. Still, up-regulation of SOCS1 mRNA levels was shown in CD4+ T cells isolated from HAM/TSP patients and asymptomatic carriers, but not from ATLL patients [50]. Furthermore, HTLV-1 Tax has been shown to induce SOCS1 expression, leading to the inhibition of RIG-I-dependent antiviral signaling, but not the JAK/STAT signaling pathways [58]. Inhibition of cytoplasmatic pattern recognition receptors such as RIG-I, has been associated with IRF3 inhibition and thus subsequent inhibition of type I interferon production. Consequently, HTLV-1-induced SOCS1 expression could counteract activation of IFN-α signaling via reduced type I interferon production. However, whereas exogenous IFN-α has been shown to increase SOCS1 expression in HeLa-cells, HTLV-1 expressed from an infectious molecular clone reduced IFN-α-induced up-regulation of SOCS1 mRNA levels [48]. Taken together, the precise correlation between HTLV-1 and SOCS1 expression and its effect on IFN-α signaling, remains unclear. Our microarray analysis revealed significant IFN-α-induced up-regulation of suppressors of cytokine signaling SOCS1, SOCS2 and SOCS3 levels in MT-4 cells, but not in MT-2 cells, although IPA pathway analysis revealed strikingly similar IFN signaling in both cell lines. Therefore, an IFN-α-induced increased SOCS1 level is not a generalized finding in HTLV-1 infection in vitro and is not by itself sufficient to define the blunted biological activity of IFN-α. HTLV-1 expression has also been reported to up-regulate IRF4 levels in HTLV-1-transformed cell lines and PBMCs of ATLL patients [59,60]. IRF4 was shown to negatively regulate type I interferon production and appeared to be associated with AZT + IFN-α antiviral resistance in ATLL patients [59-61]. Our microarray results showed no effect of IFN-α on IRF4 expression in MT-2 or MT-4 cells, although more sensitive nCounter analysis revealed slight IFN-α-induced up-regulation of IFR4 levels, which was significant in MT-4 cells, but not in MT-2 cells (p = 0.049 and p = 0.65, respectively, data not shown). Furthermore, treatment with exogenous IFN-α was able to activate IFN-α signalling to a similar extent in both cell lines (Figure 6A-B and Tables 1,2), although SOCS1 and IRF4 were significantly up-regulated in MT-4 cells only. Nevertheless, our study was limited to the broad antiviral, pro-apoptotic and antiproliferative activities of IFN-α, as well as IFN-α signaling in HTLV-1-infected cells, whereas the precise contribution of cellular factors such as SOCS1 or IRF4 has been investigated in detail in previous studies [50,58-61]. However, it should be stated that some of these studies [48,50] investigate *de novo* infection with HTLV-1 molecular clones, in contrast to stable HTLV-1 infection (this study). The latter might be closer to the in vivo situation, considering the latency of the virus in vivo and its slow molecular evolution, pointing at limited *de novo* infection [1]. In addition, there is increasing evidence that activation of multiple IFN-α signaling pathways is required to generate the antiviral, pro-apoptotic and immunomodulatory effects of IFN-α [16]. Antiviral and antiproliferative activities of IFN-α have been reported to depend on both STAT- and p38-signaling pathways [62]. Although we observed transcriptional activation of STAT1, STAT2 and downstream ISGs in both MT-2 and MT-4 cells, other important IFN-α signaling pathways, such as p38-signaling, could be affected by HTLV-1 replication, possibly explaining the absence of explicit antiviral or antiproliferative activity against HTLV-1 in vitro.

Altogether, one can assume that the in vitro antiretroviral activity and, possibly, the in vivo therapeutic success of IFN-α for both HIV-1 and HTLV-1 is determined by virus-specific factors including viral life cycle-related factors (replication, virion production) and the balance between factors blunting or stimulating IFN-α signaling pathways. For example, inhibition of HIV-1 assembly and release of virions by IFN-α has been described through the induction of ISG15, an ubiquitin-like protein [34]. Through ISG15 up-regulation, IFN-α could affect HTLV-1 assembly, which could explain the post-transcriptional inhibition of p19 secretion. On the other hand, a recent systematic screen for antiviral activity of 389 ISGs, revealed the ability of ADAR to enhance HIV-1 replication [31]. Unfortunately, no data are available on IFN signaling and/or specific ISGs levels in HIV-1/HTLV-1 co-infection, which is of significant clinical importance since several cohort studies have revealed accelerated clinical progression to AIDS and/or increased mortality in HIV-1/HTLV-1 co-infected versus HIV-1 mono-infected individuals [63-65].

## Conclusion

We found that IFN-α treatment of retrovirus-infected CD4+ T cells revealed strong anti-HIV-1 but limited anti-HTLV-1 effects. We demonstrate intact early as well as sustained IFN-α signaling in HTLV-1-infected cells, despite various reports of SOCS1- and HTLV-1 Tax-induced blunting of IFN-α signaling. We speculate that qualitative rather than quantitative differences in IFN-α signaling and downstream ISGs could determine the therapeutic success of IFN-α in vivo, depending on both host genetics and disease context. Therefore, large cohorts with long-term clinical follow-up of ATLL and HAM/TSP patients, as well as HIV-1/HTLV-1 co-infected individuals are required to identify viral and host factors specifically limiting antiretroviral activity of IFN-α against HTLV-1.

## Materials and methods

### Reagents

IFN-α2A (3x10^6^ IU/ml, a gift of Blausiegel Farmacêutica, São Paulo, Brazil) stock solutions were prepared in normal saline and working solutions in RPMI 1640 medium, supplemented with 10% heat inactivated foetal calf serum, 20 μg/ml gentamicin and 75 mM NaHCO_3_ (GIBCO® Invitrogen, Belgium). Antiviral activity of IFN-α was confirmed in a Vesicular Stomatitis Virus/WISH bioassay [66].

### Patients samples and cell lines

Diagnosis of HTLV-1 infection, HAM/TSP and ATLL was made according to published criteria [67], combining ELISA (Murex), Western blot, INNO-LIA (Innogenetics) and clinical data. Written informed consent was obtained from all participants and this study was approved by the Ethics Committees of CpqGM-FIOCRUZ and HUPES/UFBA (Salvador-Bahia, Brazil) and the Universidad Peruana Cayetano Heredia (Lima, Peru). PBMCs of 5 HAM/TSP and 6 ATLL patients (4 acute, 2 chronic) were isolated by Ficoll-Hypaque density gradient centrifugation (Sigma-Aldrich).

PBMCs and chronically HTLV-1-infected cell lines, C8166, MT-2 and MT-4 [68-70], were cultured in supplemented RPMI 1640 medium. C8166 cells were obtained from the Medical Research Council, UK), MT-2 and MT-4 cells were from Harada *et al.*[71]. For varying time spans, PBMCs (2 x 10^6^ cells/ml) and HTLV-1-infected cell lines (2 x 10^5^ cells/ml) were treated in the absence or presence of IFN-α (10 - 10^4^ IU/ml) followed by flow cytometric analysis of HTLV-1-infected cell lines. Given that MT-4 cells are highly susceptible to HIV-1 infection and have been successfully used as a drug screening model for the detection of anti-HIV compounds [53], MT-4 cells (6 x 10^5^ cells/ml) were infected with HIV-1 III_B_ strain [72] and cultured for 5 days in the absence or presence of varying concentrations of IFN-α (0.01 - 10^4^ IU/ml). Hereby, antiviral activity of IFN-α was measured via the 3-(4,5-dimethylthiazol-2-yl)-2,5-diphenyltetrazolium bromide (MTT) cell viability staining assay [53,73]. In parallel, HTLV-1-mono-infected MT-4 cells (6 x 10^5^ cells/ml) were cultured for 5 days in the absence or presence of varying concentrations of IFN-α (10 - 10^4^ IU/ml). Cell viability was quantified by both MTT assay and trypan blue dye exclusion protocols.

### Flow cytometry assay

At different time points after IFN-α treatment, cell lines were collected and DNA content (Hoechst 33342), cell death-associated (active-caspase-3, surface Fas) and cell proliferation-associated (PCNA) markers were quantified by flow cytometry (BD Biosciences). Briefly, cells were fixed in Cytofix buffer (BD Biosciences) for 10 minutes at 37°C. Cell pellets were permeabilized in 100% ice-cold methanol for 30 minutes. Cells were then washed twice in 1x PBA (phosphate-buffered saline + bovine serum albumin + NaN_3_) and incubated with fluorescence-labelled monoclonal antibodies at room temperature (APC mouse IgG1, FITC mouse IgG2a, PE mouse IgG2a, APC anti-Fas, FITC anti-PCNA, PE anti-active Caspase-3). After 30 minutes, cells were washed twice in 1 x PBA and stained with Hoechst 33342. Cell populations and debris were defined based on morphology via forward- versus side-scatter plots and 10.000-100.000 events were acquired per sample.

### HIV-1 p24 and HTLV-1 p19 quantification

HIV-1 capsid protein p24 and HTLV-1 matrix protein p19 were quantified in cell-free supernatant of MT-4 and MT-2 cells, using HIV-1 p24 Core Profile ELISA kit (PerkinElmer) and RetroTek HTLV-I/II p19 Antigen ELISA kit (ZeptoMetrix), respectively. After 48–72 hours of IFN-α treatment (1000 IU/ml), HTLV-1 p19 levels were quantified in cell-free supernatant of HAM/TSP and ATLL PBMCs.

### Microarray analysis

Total RNA was extracted from MT-2 and MT-4 cells treated in the absence or presence of IFN-α (1000 IU/ml) at 6 hours, using RNeasy kit according to the manufacturer’s protocol (QIAgen Benelux B.V., Venlo, the Netherlands). Agilent Whole Human Genome microarray analysis was performed by the VIB MicroArray Facility (Leuven, Belgium). Data were analysed using the Agilent Feature Extraction Software version 10.1.1.1 and 10.5.1.1. Briefly, the intensities of the fluorescent probes Cy3 and Cy5, representing the transcription values, were log_2_-transformed and normalized by quantile normalization using the R package preprocessCore [74]. The contrasts in expression between IFN-α-treated and untreated cells at 6 hours of stimulation were estimated using the Limma package from Bioconductor (http://www.bioconductor.org). For the selection of differentially transcribed genes, a fold-change cut-off of two (*i.e.* an absolute log_2_-ratio larger than 1) was combined with a p-value cut-off of p < 0.05 when corrected for multiple testing. GEO submission of microarray data is detailed in Additional file 2.

### Ingenuity pathway analysis

The Ingenuity Pathway Analysis (IPA) program was used to perform a pathway/function level analysis on genes resulting from the microarray analysis for both MT-2 and MT-4 (IPA version 9.0, Build 116623, Content version 3211, Ingenuity Systems, Red Wood City, CA). Hereby, uncorrected p-values and absolute fold-changes were used with cut-offs of p < 0.001 and fold-change value of 2 (up or down), respectively. Based on a scientific literature database, genes were sorted into gene networks and canonical pathways and significantly overrepresented pathways were identified. The maximum number of networks to be generated was set to 25, with a maximum number of 35 molecules per network. The signaling pathways and networks in Figure 3 were generated through the use of IPA (Ingenuity Systems, http://www.ingenuity.com).

### nCounter analysis

Total RNA was extracted from MT-2 and MT-4 treated in the absence and presence of IFN-α (1000 IU/ml) at 2, 6 and 48 hours, using RNeasy kit according to the manufacturer’s protocol (QIAgen Benelux B.V., Venlo, the Netherlands). nCounter^TM^ (NanoString Technologies, Seattle, United States) analysis was performed at the VIB MicroArray Facility (Leuven, Belgium), based on direct molecular bar-coding of target RNA transcripts and digital detection [56]. Through the use of colour-coded probe pairs, without the use of reverse transcriptase nor amplification, mRNA transcripts of HTLV-1 viral genes and specific cellular genes, including housekeeping genes for normalization, were quantified in MT-2 and MT-4 cells after 2, 6 and 48 hours of IFN-α treatment (HTLV-1 Tax/Rex, Gag, protease, polymerase, gp46, gp21, NC_001436.1; HTLV-1 p13, p12, p30, AB513134; HTLV-1 HBZ, DQ273132; STAT1, NM_007315.2; STAT2, NM_005419.2; STAT3, NM_139276.2; CD69, NM_001781.1).

### Statistical analysis

Statistical analysis was performed with GraphPad Prism 5 software. Parametric t-tests were used and p < 0.05 was considered significant.

In microarray analysis, a moderated *t*-test was used, as implemented in the Limma package, to test whether a contrast was significantly different from zero. To verify the false discovery rate, p-values were corrected for multiple testing with Benjamini-Hochberg.

## Competing interests

The author(s) declare that they have no competing interests.

## Authors’ contributions

JVW conceived this project and designed the experiments. BM carried out the experiments with cell lines and wrote the manuscript. GL, RK and JVW performed the experiments with patient samples. CP and AV participated in the study design and helped to draft the manuscript. MT, EG, AB, LF and BGC provided the patient samples. All authors read and approved the final manuscript.

## Supplementary Material

Additional file 1 **Early and late effects of IFN-α on mRNA levels of STAT and HTLV-1 genes.** The HTLV-1-infected CD4+ T cell line MT-4 was treated for 2 and 48 hours with no treatment (NT) or IFN-α (1000 IU/ml). mRNA levels of cellular and viral genes were assessed via nCounter analysis. (A) STAT1 and STAT2 mRNA levels are represented for MT-4 cells after 2 hours of treatment. mRNA levels of (B) HTLV-1 viral genes and (C) STAT1 and STAT2 cellular genes are represented after 48 hours of treatment. mRNA levels (fM) are shown in the *y*-axis, whereas the treatment conditions are shown in the *x*-axis. Parametric t-tests were used with p-values indicated by asterisks (^*^ < 0.05, ^**^ < 0.01 and ^***^ < 0.001). Click here for file

Additional file 2 GEO submission microarray data.doc. Click here for file

## References

[B1] Verdonck K Gonzalez E Van Dooren S Vandamme AM Vanham G Gotuzzo E Human T-lymphotropic virus 1: recent knowledge about an ancient infection Lancet Infect Dis 2007 7 4 266 281 10.1016/S1473-3099(07)70081-6 17376384

[B2] Kallings LO The first postmodern pandemic: 25 years of HIV/AIDS J Intern Med 2008 263 3 218 243 10.1111/j.1365-2796.2007.01910.x 18205765

[B3] De Clercq E Antiretroviral drugs Curr Opin Pharmacol 2010 10 5 507 515 10.1016/j.coph.2010.04.011 20471318

[B4] Gessain A Barin F Vernant JC Gout O Maurs L Calender A de The G Antibodies to human T-lymphotropic virus type-I in patients with tropical spastic paraparesis Lancet 1985 2 8452 407 410 2863442 10.1016/s0140-6736(85)92734-5

[B5] Tajima K Cartier L Epidemiological features of HTLV-I and adult T cell leukemia Intervirology 1995 38 3–4 238 246 8682622 10.1159/000150438

[B6] Tsukasaki K Hermine O Bazarbachi A Ratner L Ramos JC Harrington W O'Mahony D, Janik JE, Bittencourt AL, Taylor GP, Yamaguchi K, Utsunomiya A, Tobinai K, Watanabe T: **Definition, prognostic factors, treatment, and response criteria of adult T-cell leukemia-lymphoma: a proposal from an international consensus meeting** J Clin Oncol 2009 27 3 453 459 19064971 10.1200/JCO.2008.18.2428 PMC2737379

[B7] Bazarbachi A Plumelle Y Carlos Ramos J Tortevoye P Otrock Z Taylor G Gessain A Harrington W Panelatti G Hermine O Meta-analysis on the use of zidovudine and interferon-alfa in adult T-cell leukemia/lymphoma showing improved survival in the leukemic subtypes J Clin Oncol 2010 28 27 4177 4183 10.1200/JCO.2010.28.0669 20585095

[B8] Osame M Usuku K Izumo S Ijichi N Amitani H Igata A Matsumoto M Tara M HTLV-I associated myelopathy, a new clinical entity Lancet 1986 1 8488 1031 1032 2871307 10.1016/s0140-6736(86)91298-5

[B9] Izumo S Umehara F Osame M HTLV-I-associated myelopathy Neuropathology 2000 20 Suppl S65 S68 11037191 10.1046/j.1440-1789.2000.00320.x

[B10] Nakagawa M Nakahara K Maruyama Y Kawabata M Higuchi I Kubota H Izumo S Arimura K Osame M Therapeutic trials in 200 patients with HTLV-I-associated myelopathy/tropical spastic paraparesis J Neurovirol 1996 2 5 345 355 10.3109/13550289609146899 8912211

[B11] Vallinoto AC Santana BB dos Santos EL Santo RR Hermes RB Sousa RC Cayres-Vallinoto I Machado LF Ishak MO Ishak R FAS-670A/G single nucleotide polymorphism may be associated with human T lymphotropic virus-1 infection and clinical evolution to TSP/HAM Virus Res 2012 163 1 178 182 10.1016/j.virusres.2011.09.015 21971214

[B12] Farre L Bittencourt AL Silva-Santos G Almeida A Silva AC Decanine D Soares GM Alcantara LC Van Dooren S Galvão-Castro B Vandamme AM Van Weyenbergh J Fas 670 promoter polymorphism is associated to susceptibility, clinical presentation, and survival in adult T cell leukemia J Leukoc Biol 2008 83 1 220 222 17962369 10.1189/jlb.0407198

[B13] Lane HC Davey V Kovacs JA Feinberg J Metcalf JA Herpin B Walker R Deyton L Davey RT Falloon J Interferon-alpha in patients with asymptomatic human immunodeficiency virus (HIV) infection. A randomized, placebo-controlled trial Ann Intern Med 1990 112 11 805 811 1971503 10.7326/0003-4819-112-11-805

[B14] Isaacs A Lindenmann J Virus interference I. The interferon. Proc R Soc Lond B Biol Sci 1957 147 927 258 267 10.1098/rspb.1957.0048 26297790

[B15] Takaoka A Yanai H Interferon signalling network in innate defence Cell Microbiol 2006 8 6 907 922 10.1111/j.1462-5822.2006.00716.x 16681834

[B16] Platanias LC Mechanisms of type-I- and type-II-interferon-mediated signalling Nat Rev Immunol 2005 5 5 375 386 10.1038/nri1604 15864272

[B17] Taniguchi T Takaoka A The interferon-alpha/beta system in antiviral responses: a multimodal machinery of gene regulation by the IRF family of transcription factors Curr Opin Immunol 2002 14 1 111 116 10.1016/S0952-7915(01)00305-3 11790540

[B18] Trinchieri G Type I interferon: friend or foe? J Exp Med 2010 207 10 2053 2063 10.1084/jem.20101664 20837696 PMC2947062

[B19] de Veer MJ Holko M Frevel M Walker E Der S Paranjape JM Silverman RH Williams BR Functional classification of interferon-stimulated genes identified using microarrays J Leukoc Biol 2001 69 6 912 920 11404376

[B20] Goujon C Malim MH Characterization of the alpha interferon-induced postentry block to HIV-1 infection in primary human macrophages and T cells J Virol 2010 84 18 9254 9266 10.1128/JVI.00854-10 20610724 PMC2937661

[B21] Borden EC Sen GC Uze G Silverman RH Ransohoff RM Foster GR Stark GR Interferons at age 50: past, current and future impact on biomedicine Nat Rev Drug Discov 2007 6 12 975 990 10.1038/nrd2422 18049472 PMC7097588

[B22] King JK Yeh SH Lin MW Liu CJ Lai MY Kao JH Chen DS Chen PJ Genetic polymorphisms in interferon pathway and response to interferon treatment in hepatitis B patients: A pilot study Hepatology 2002 36 6 1416 1424 12447867 10.1053/jhep.2002.37198

[B23] Pflugheber J Fredericksen B Sumpter R Wang C Ware F Sodora DL Gale M Regulation of PKR and IRF-1 during hepatitis C virus RNA replication Proc Natl Acad Sci USA 2002 99 7 4650 4655 10.1073/pnas.062055699 11904369 PMC123702

[B24] Golomb HM Hairy cell leukemia: treatment successes in the past 25 years J Clin Oncol 2008 26 16 2607 2609 10.1200/JCO.2007.15.7420 18509168

[B25] Fried MW Shiffman ML Reddy KR Smith C Marinos G Goncales FL Haussinger D Diago M Carosi G Dhumeaux D Craxi A Lin A Hoffman J Yu J Peginterferon alfa-2a plus ribavirin for chronic hepatitis C virus infection N Engl J Med 2002 347 13 975 982 10.1056/NEJMoa020047 12324553

[B26] Ferrantini M Capone I Belardelli F Interferon-alpha and cancer: mechanisms of action and new perspectives of clinical use Biochimie 2007 89 6–7 884 893 17532550 10.1016/j.biochi.2007.04.006

[B27] Moschos S Kirkwood JM Present role and future potential of type I interferons in adjuvant therapy of high-risk operable melanoma Cytokine Growth Factor Rev 2007 18 5–6 451 458 17693125 10.1016/j.cytogfr.2007.06.020

[B28] van Boxel-Dezaire AH Rani MR Stark GR Complex modulation of cell type-specific signaling in response to type I interferons Immunity 2006 25 3 361 372 10.1016/j.immuni.2006.08.014 16979568

[B29] Chevaliez S Pawlotsky JM Interferon-based therapy of hepatitis C Adv Drug Deliv Rev 2007 59 12 1222 1241 10.1016/j.addr.2007.07.002 17869375

[B30] Pestka S Krause CD Walter MR Interferons, interferon-like cytokines, and their receptors Immunol Rev 2004 202 8 32 10.1111/j.0105-2896.2004.00204.x 15546383

[B31] Schoggins JW Wilson SJ Panis M Murphy MY Jones CT Bieniasz P Rice CM A diverse range of gene products are effectors of the type I interferon antiviral response Nature 2011 472 7344 481 485 10.1038/nature09907 21478870 PMC3409588

[B32] Korth MJ Taylor MD Katze MG Interferon inhibits the replication of HIV-1, SIV, and SHIV chimeric viruses by distinct mechanisms Virology 1998 247 2 265 273 10.1006/viro.1998.9249 9705919

[B33] Hartshorn KL Neumeyer D Vogt MW Schooley RT Hirsch MS Activity of interferons alpha, beta, and gamma against human immunodeficiency virus replication in vitro AIDS Res Hum Retroviruses 1987 3 2 125 133 10.1089/aid.1987.3.125 3113463

[B34] Okumura A Lu G Pitha-Rowe I Pitha PM Innate antiviral response targets HIV-1 release by the induction of ubiquitin-like protein ISG15 Proc Natl Acad Sci USA 2006 103 5 1440 1445 10.1073/pnas.0510518103 16434471 PMC1360585

[B35] Agy MB Acker RL Sherbert CH Katze MG Interferon treatment inhibits virus replication in HIV-1- and SIV-infected CD4+ T-cell lines by distinct mechanisms: evidence for decreased stability and aberrant processing of HIV-1 proteins Virology 1995 214 2 379 386 10.1006/viro.1995.0047 8553538

[B36] Baca-Regen L Heinzinger N Stevenson M Gendelman HE Alpha interferon-induced antiretroviral activities: restriction of viral nucleic acid synthesis and progeny virion production in human immunodeficiency virus type 1-infected monocytes J Virol 1994 68 11 7559 7565 7933143 10.1128/jvi.68.11.7559-7565.1994 PMC237202

[B37] Coccia EM Krust B Hovanessian AG Specific inhibition of viral protein synthesis in HIV-infected cells in response to interferon treatment J Biol Chem 1994 269 37 23087 23094 7521875

[B38] Yamamoto JK Barre-Sinoussi F Bolton V Pedersen NC Gardner MB Human alpha- and beta-interferon but not gamma- suppress the in vitro replication of LAV, HTLV-III, and ARV-2 J Interferon Res 1986 6 2 143 152 10.1089/jir.1986.6.143 2425014

[B39] Mandl JN Barry AP Vanderford TH Kozyr N Chavan R Klucking S Barrat FJ Coffman RL Staprans SI Feinberg MB Divergent TLR7 and TLR9 signaling and type I interferon production distinguish pathogenic and nonpathogenic AIDS virus infections Nat Med 2008 14 10 1077 1087 10.1038/nm.1871 18806803

[B40] Rotger M Dang KK Fellay J Heinzen EL Feng S Descombes P Shianna KV Ge D Gunthard HF Goldstein DB Telenti A Genome-wide mRNA expression correlates of viral control in CD4+ T-cells from HIV-1-infected individuals PLoS Pathog 2010 6 2 e1000781 10.1371/journal.ppat.1000781 20195503 PMC2829051

[B41] von Sydow M Sonnerborg A Gaines H Strannegard O Interferon-alpha and tumor necrosis factor-alpha in serum of patients in various stages of HIV-1 infection AIDS Res Hum Retroviruses 1991 7 4 375 380 10.1089/aid.1991.7.375 1906289

[B42] Gringeri A Musicco M Hermans P Bentwich Z Cusini M Bergamasco A Santagostino E Burny A Bizzini B Zagury D Active anti-interferon-alpha immunization: a European-Israeli, randomized, double-blind, placebo-controlled clinical trial in 242 HIV-1–infected patients (the EURIS study) J Acquir Immune Defic Syndr Hum Retrovirol 1999 20 4 358 370 10.1097/00042560-199904010-00006 10096580

[B43] Herbeuval JP Shearer GM HIV-1 immunopathogenesis: how good interferon turns bad Clin Immunol 2007 123 2 121 128 10.1016/j.clim.2006.09.016 17112786 PMC1930161

[B44] Haas DW Lavelle J Nadler JP Greenberg SB Frame P Mustafa N St Clair M McKinnis R Dix L Elkins M Rooney J A randomized trial of interferon alpha therapy for HIV type 1 infection AIDS Res Hum Retroviruses 2000 16 3 183 190 10.1089/088922200309278 10710206

[B45] Asmuth DM Murphy RL Rosenkranz SL Lertora JJ Kottilil S Cramer Y Chan ES Schooley RT Rinaldo CR Thielman N Li XD Wahl SM Shore J Janik J Lempicki RA Simpson Y Pollard RB Safety, tolerability, and mechanisms of antiretroviral activity of pegylated interferon Alfa-2a in HIV-1-monoinfected participants: a phase II clinical trial J Infect Dis 2010 201 11 1686 1696 10.1086/652420 20420510 PMC2946345

[B46] Hatzakis A Gargalianos P Kiosses V Lazanas M Sypsa V Anastassopoulou C Vigklis V Sambatakou H Botsi C Paraskevis D Stalgis C Low-dose IFN-alpha monotherapy in treatment-naive individuals with HIV-1 infection: evidence of potent suppression of viral replication J Interferon Cytokine Res 2001 21 10 861 869 10.1089/107999001753238114 11710999

[B47] Feng X Heyden NV Ratner L Alpha interferon inhibits human T-cell leukemia virus type 1 assembly by preventing Gag interaction with rafts J Virol 2003 77 24 13389 13395 10.1128/JVI.77.24.13389-13395.2003 14645593 PMC296084

[B48] Feng X Ratner L Human T-cell leukemia virus type 1 blunts signaling by interferon alpha Virology 2008 374 1 210 216 10.1016/j.virol.2007.12.036 18234266 PMC2373983

[B49] Zhang J Yamada O Kawagishi K Araki H Yamaoka S Hattori T Shimotohno K Human T-cell leukemia virus type 1 Tax modulates interferon-alpha signal transduction through competitive usage of the coactivator CBP/p300 Virology 2008 379 2 306 313 10.1016/j.virol.2008.06.035 18678383

[B50] Oliere S Hernandez E Lezin A Arguello M Douville R Nguyen TL Olindo S Panelatti G Kazanji M Wilkinson P Sekaly RP Cesaire R Hiscott J HTLV-1 evades type I interferon antiviral signaling by inducing the suppressor of cytokine signaling 1 (SOCS1) PLoS Pathog 2010 6 11 e1001177 10.1371/journal.ppat.1001177 21079688 PMC2973829

[B51] Gill PS Harrington W Kaplan MH Ribeiro RC Bennett JM Liebman HA Bernstein-Singer M Espina BM Cabral L Allen S Treatment of adult T-cell leukemia-lymphoma with a combination of interferon alfa and zidovudine N Engl J Med 1995 332 26 1744 1748 10.1056/NEJM199506293322603 7760890

[B52] Izumo S Goto I Itoyama Y Okajima T Watanabe S Kuroda Y Araki S Mori M Nagataki S Matsukura S Akamine T Nakagawa M Yamamoto I Osame M Interferon-alpha is effective in HTLV-I-associated myelopathy: a multicenter, randomized, double-blind, controlled trial Neurology 1996 46 4 1016 1021 10.1212/WNL.46.4.1016 8780082

[B53] Pannecouque C Daelemans D De Clercq E Tetrazolium-based colorimetric assay for the detection of HIV replication inhibitors: revisited 20 years later Nat Protoc 2008 3 3 427 434 10.1038/nprot.2007.517 18323814

[B54] Moens B Decanine D Menezes SM Khouri R Silva-Santos G Lopez G Alvarez C Talledo M Gotuzzo E de Almeida Kruschewsky R Galvão-Castro B Vandamme AM Van Weyenbergh J Ascorbic Acid Has Superior *Ex Vivo* Antiproliferative, Cell Death-Inducing and Immunomodulatory Effects over IFN-α in HTLV-1-Associated Myelopathy PLoS Negl Trop Dis 2012 6 7 e1729 10.1371/journal.pntd.0001729 22848768 PMC3404116

[B55] Tattermusch S Skinner JA Chaussabel D Banchereau J Berry MP McNab FW O'Garra A Taylor GP Bangham CR Systems Biology Approaches Reveal a Specific Interferon-Inducible Signature in HTLV-1 Associated Myelopathy PLoS Pathog 2012 8 1 e1002480 10.1371/journal.ppat.1002480 22291590 PMC3266939

[B56] Geiss GK Bumgarner RE Birditt B Dahl T Dowidar N Dunaway DL Fell HP Ferree S George RD Grogan T James JJ Maysuria M Mitton JD Oliveri P Osborn JL Peng T Ratcliffe AL Webster PJ Davidson EH Hood L Dimitrov K Direct multiplexed measurement of gene expression with color-coded probe pairs Nat Biotechnol 2008 26 3 317 325 10.1038/nbt1385 18278033

[B57] Chakrabarti A Jha BK Silverman RH New insights into the role of RNase L in innate immunity J Interferon Cytokine Res 2011 31 1 49 57 10.1089/jir.2010.0120 21190483 PMC3021357

[B58] Charoenthongtrakul S Zhou Q Shembade N Harhaj NS Harhaj EW Human T cell leukemia virus type 1 Tax inhibits innate antiviral signaling via NF-kappaB-dependent induction of SOCS1 J Virol 2011 85 14 6955 6962 10.1128/JVI.00007-11 21593151 PMC3126571

[B59] Sharma S Grandvaux N Mamane Y Genin P Azimi N Waldmann T Hiscott J Regulation of IFN regulatory factor 4 expression in human T cell leukemia virus-I-transformed T cells J Immunol 2002 169 6 3120 3130 12218129 10.4049/jimmunol.169.6.3120

[B60] Imaizumi Y Kohno T Yamada Y Ikeda S Tanaka Y Tomonaga M Matsuyama T Possible involvement of interferon regulatory factor 4 (IRF4) in a clinical subtype of adult T-cell leukemia Jpn J Cancer Res 2001 92 12 1284 1292 10.1111/j.1349-7006.2001.tb02151.x 11749693 PMC5926682

[B61] Ramos JC Ruiz P Ratner L Reis IM Brites C Pedroso C Byrne GE Toomey NL Andela V Harhaj EW Lossos IS Harrington WJ IRF-4 and c-Rel expression in antiviral-resistant adult T-cell leukemia/lymphoma Blood 2007 109 7 3060 3068 17138822 10.1182/blood-2006-07-036368 PMC1852214

[B62] Platanias LC The p38 mitogen-activated protein kinase pathway and its role in interferon signaling Pharmacol Ther 2003 98 2 129 142 10.1016/S0163-7258(03)00016-0 12725866

[B63] Pedroso C Netto EM Weyll N Brites C Coinfection by HIV-1 and human lymphotropic virus type 1 in Brazilian children is strongly associated with a shorter survival time J Acquir Immune Defic Syndr 2011 57 Suppl 3 S208 S211 21857320 10.1097/QAI.0b013e31821e9baf

[B64] Schechter M Harrison LH Halsey NA Trade G Santino M Moulton LH Quinn TC Coinfection with human T-cell lymphotropic virus type I and HIV in Brazil. Impact on markers of HIV disease progression JAMA 1994 271 5 353 357 10.1001/jama.1994.03510290035033 7904317

[B65] Brites C Sampalo J Oliveira A HIV/human T-cell lymphotropic virus coinfection revisited: impact on AIDS progression AIDS Rev 2009 11 1 8 16 19290030

[B66] Van Weyenbergh J Lipinski P Abadie A Chabas D Blank U Liblau R Wietzerbin J Antagonistic action of IFN-beta and IFN-gamma on high affinity Fc gamma receptor expression in healthy controls and multiple sclerosis patients J Immunol 1998 161 3 1568 1574 9686625

[B67] Carneiro-Proietti AB Catalan-Soares BC Castro-Costa CM Murphy EL Sabino EC Hisada M Galvao-Castro B Alcantara LC Remondegui C Verdonck K Proietti FA HTLV in the Americas: challenges and perspectives Rev Panam Salud Publica 2006 19 1 44 53 10.1590/S1020-49892006000100007 16536938

[B68] Miyoshi I Yoshimoto S Kubonishi I Taguchi H Shiraishi Y Ohtsuki Y Akagi T Transformation of normal human cord lymphocytes by co-cultivation with a lethally irradiated human T-cell line carrying type C virus particles Gann 1981 72 6 997 998 6281121

[B69] Salahuddin SZ Markham PD Wong-Staal F Franchini G Kalyanaraman VS Gallo RC Restricted expression of human T-cell leukemia–lymphoma virus (HTLV) in transformed human umbilical cord blood lymphocytes Virology 1983 129 1 51 64 10.1016/0042-6822(83)90395-1 6412453

[B70] Miyoshi I Kubonishi I Yoshimoto S Shiraishi Y A T-cell line derived from normal human cord leukocytes by co-culturing with human leukemic T-cells Gann 1981 72 6 978 981 6281119

[B71] Harada S Koyanagi Y Yamamoto N Infection of HTLV-III/LAV in HTLV-I-carrying cells MT-2 and MT-4 and application in a plaque assay Science 1985 229 4713 563 566 10.1126/science.2992081 2992081

[B72] Popovic M Sarngadharan MG Read E Gallo RC Detection, isolation, and continuous production of cytopathic retroviruses (HTLV-III) from patients with AIDS and pre-AIDS Science 1984 224 4648 497 500 10.1126/science.6200935 6200935

[B73] Pauwels R Balzarini J Baba M Snoeck R Schols D Herdewijn P Desmyter J De Clercq E Rapid and automated tetrazolium-based colorimetric assay for the detection of anti-HIV compounds J Virol Methods 1988 20 4 309 321 10.1016/0166-0934(88)90134-6 2460479

[B74] Bolstad BM Irizarry RA Astrand M Speed TP A comparison of normalization methods for high density oligonucleotide array data based on variance and bias Bioinformatics 2003 19 2 185 193 10.1093/bioinformatics/19.2.185 12538238

