# Supplementary material for: Simultaneous RNA quantification of human and retroviral genomes reveals intact interferon signaling in HTLV-1-infected CD4+ T cell lines
Source: Virol J. 2012 Aug 23;9:171. doi: 10.1186/1743-422X-9-171 (PMC3492208; doi:10.1186/1743-422X-9-171)
Supplement: Additional file 2 — GEO submission microarray data.doc. [file 1743-422X-9-171-S2.doc]

**Gene Expression Omnibus submission of microarray data**

Microarray data of MT-2 and MT-4 cells treated with IFN-α or other candidate drugs were submitted to Gene Expression Omnibus, according to MIAME guidelines, with following accession number and reviewers link:

GSE34870

[http://www.ncbi.nlm.nih.gov/geo/query/acc.cgi?token=xhozlyweoiwquby&acc=GSE34870](https://legacy-owa.groupware.kuleuven.be/owa/redir.aspx?C=cfbc13e7e7fa481684bc8d9240fc36e7&URL=http%3A%2F%2Fwww.ncbi.nlm.nih.gov%2Fgeo%2Fquery%2Facc.cgi%3Ftoken%3Dxhozlyweoiwquby%26acc%3DGSE34870)
